# Mechanical Properties and Bioactivity of Poly(Lactic Acid) Composites Containing Poly(Glycolic Acid) Fiber and Hydroxyapatite Particles

**DOI:** 10.3390/nano11010249

**Published:** 2021-01-18

**Authors:** Han-Seung Ko, Sangwoon Lee, Doyoung Lee, Jae Young Jho

**Affiliations:** School of Chemical and Biological Engineering, Seoul National University, Seoul 08826, Korea; khs88@snu.ac.kr (H.-S.K.); tttyyy0403@snu.ac.kr (S.L.); dyoung94@snu.ac.kr (D.L.)

**Keywords:** poly(lactic acid), polymer composite, hydroxyapatite, polymer grafting, poly(glycolic acid) fiber, mechanical properties

## Abstract

To enhance the mechanical strength and bioactivity of poly(lactic acid) (PLA) to the level that can be used as a material for spinal implants, poly(glycolic acid) (PGA) fibers and hydroxyapatite (HA) were introduced as fillers to PLA composites. To improve the poor interface between HA and PLA, HA was grafted by PLA to form HA-g-PLA through coupling reactions, and mixed with PLA. The size of the HA particles in the PLA matrix was observed to be reduced from several micrometers to sub-micrometer by grafting PLA onto HA. The tensile and flexural strength of PLA/HA-g-PLA composites were increased compared with those of PLA/HA, apparently due to the better dispersion of HA and stronger interfacial adhesion between the HA and PLA matrix. We also examined the effects of the length and frequency of grafted PLA chains on the tensile strength of the composites. By the addition of unidirectionally aligned PGA fibers, the flexural strength of the composites was greatly improved to a level comparable with human compact bone. In the bioactivity tests, the growth of apatite on the surface was fastest and most uniform in the PLA/PGA fiber/HA-g-PLA composite.

## 1. Introduction

Mechanical strength and bioactivity have been cited as the two most important properties that materials for spinal implants should have. Spinal implant materials based on metals like titanium and magnesium have dominated the market due to their excellent mechanical properties and processability. Metal implants, however, have caused the stress shielding effect, a phenomenon of low load on lower-modulus material around the implant. This has been problematic in the implants, reducing the density of the regenerated bones [1]. Since polymers could avoid the stress shielding effect due to their lower modulus than metals, the use of biodegradable polymers as implant materials has been sought [2]. In addition, an implant based on biodegradable polymers might not require surgery to remove the implant after bone growth. Aliphatic polyesters including polycaprolactone (PCL), poly(lactic acid) (PLA), and poly(glycolic acid) (PGA) are biodegradable polymers that were considered for this application [3,4]. Among these, PCL lacked mechanical strength to be used as an implant material [5]. While PGA exhibited good mechanical properties comparable to engineering plastics, the biodegradation rate of PGA was too high [6]. This could cause the implant to be degraded before the bone was fully regenerated. As PLA showed a decent balance between mechanical properties and biodegradation rate, many studies have employing PLA as the spinal implant material have been conducted [7,8,9,10]. However, the low bioactivity and the mechanical strength lower than that of human spine bone have limited the actual application of PLA in spinal implants [11].

In order to enhance the bioactivity of PLA, PLA composites containing bioceramics such as dicalcium phosphate anhydrate, calcium metaphosphate, tricalcium phosphate, and hydroxyapatite (HA) have been developed [12,13,14,15]. It was observed that the cells involved in the bone formation proliferated more in the PLA/bioceramics composites than in PLA resin alone [12,14,15]. Since HA was structurally very similar to human bone components, many researchers have focused on PLA/HA composites [16]. However, HA has easily aggregated in composites because the surface energy of HA was much higher than that of PLA [17]. Agglomeration of HA led to the early failure of the composites, lowering the mechanical strength of the composites than that of PLA resin. The introduction of poly(acrylic acid) as a compatibilizer led to better dispersion of HA in the PLA matrix [18]. However, the tensile strength of the composites was not improved because the interfacial adhesion between the PLA matrix and HA was still poor.

The interfacial adhesion between PLA and HA has been improved by grafting PLA to HA through ring-opening polymerization (ROP) of lactide on the HA surface [19,20]. However, the amount of grafting was small because the reactivity of the surface –OH group of HA to the ROP of lactide was low. Due to the small amount of grafted PLA, the tensile strength of the composites increased by only 4% compared to that of PLA resin. Therefore, a larger amount of PLA needed to be grafted onto HA to obtain a composite with stronger interfacial adhesion and higher mechanical strength. Also, the mechanical strength of the composites was greatly affected by the interface between the filler and the matrix. However, the factors that could affect the interface, such as quantitative analysis of grafted PLA and the control of the grafting conditions, have not been specifically investigated in previous studies.

Even with the use of PLA-grafted HA, the level of mechanical strength of the PLA/HA composites was not expected to be much higher than that of PLA resin. To attain the level of mechanical strength comparable to that of human bone, an additional reinforcement was required. It has been well established that the addition of fibrous filler to polymers greatly enhanced the mechanical properties of the composites [21]. PLA composites containing various fibers including carbon fiber, carbon nanotube, natural fiber, PLA fiber, and PGA fiber have been developed [22,23,24,25,26]. Carbon fibers or carbon nanotubes were not considered to be appropriate in implant material, as they are not biodegradable. Biodegradable natural fiber had the disadvantage of poor interfacial adhesion with PLA matrix due to their polar functional groups. In the composites containing PLA fibers, the interfacial adhesion between PLA matrix and PLA fiber was strong. However, it seemed difficult to set the processing temperature and time, because the difference in melting point between the two was too small. The melting point of PGA fiber was higher than that of PLA matrix, so the solid phase of the filler could be retained even in the melting process of composite fabrication. Therefore, PGA fiber seemed to be appropriate for use as the reinforcing fiber in the PLA composites. It was expected that rapid biodegradation of PGA fibers would be avoided by covering the PGA fibers with a PLA matrix in the composites. In addition, we predicted that the mechanical properties of the composites could be significantly improved by introducing PGA fibers into the composites in a unidirectionally aligned state.

In the present study, we attempted to develop PLA composites containing both HA and aligned PGA fiber to improve the bioactivity and the mechanical properties at the same time. PLA was grafted onto HA to improve the dispersion of HA and the interfacial adhesion between HA and PLA matrix. The amount, length, and frequency of the grafted PLA chains were controlled through the coupling agents, and the effect on the mechanical strength of the composites was investigated. The bioactivity of the composites was also examined by measuring the rate and amount of apatite growth on the surface of the composites.

## 2. Materials and Methods

### 2.1. Materials

PLA used in this study was commercial product of Natureworks (Minnetonka, MN, USA) with the trade name of 2003D. This PLA contained 4.3 wt% of d-isomer [27]. HA was purchased from Nanjing Emperor Nanomaterials (Nanjing, China) under the trade name of NHAP04 in rod shape with width of 20 nm and length of 100 nm. PGA fiber was purchased from Meta Biomed (Chungcheongbuk-do, Korea). Hexamethylene diisocyanate (HMDI), dibutyltindilaurate (DBTDL), ethylene glycol (EG), and stannous 2-ethylhexanoate (Sn(Oct)_2_) were purchased from MilliporeSigma (Burlington, MA, USA). *N*,*N*-dimethylformamide (DMF), chloroform, p-xylene, and ethanol were purchased from Daejung Chemicals (Gyeonggi-do, Korea). Lactide was purchased from Tokyo Chemical Industry (Tokyo, Japan) as l-lactide with a purity of 98%.

### 2.2. Surface Modification of HA

HA (2 g) in DMF (100 mL) was sonicated and stirred under nitrogen at room temperature. HMDI (0.2 mL, 1.25 mmol) and DBTDL (7.9 mg, 1 mol% of HMDI) were added to the suspension. The reaction continued with stirring at room temperature for 6 h. Excess amount of EG (1.4 mL, 25.0 mmol, 20 times of HMDI) was added to the HMDI-grafted HA (HA-HMDI) mixture, and the additional reaction was kept overnight with stirring. After the reaction, EG-grafted HA-HMDI (HA-HMDI-EG) was separated by centrifugation at 10,000 rpm and washed with DMF for three times and chloroform for two times to completely remove the reactants and solvent. The HA-HMDI-EG was dried at 50 °C for 24 h in a vacuum oven.

HA-HMDI-EG (2 g) in p-xylene (100 mL) was sonicated at room temperature. Lactide (2 g, 13.88 mmol) in p-xylene (50 mL) was stirred at 80 °C. The HA-HMDI-EG suspension was added into the lactide solution, and the mixture was stirred under nitrogen protection for 1 h. Sn(Oct)_2_ (25 mg, 0.06 mmol) was added to the mixture, and the reaction continued with stirring at 120 °C for 18 h. After the reaction, PLA-grafted HA-HMDI-EG (HA-g-PLA) was separated by centrifugation at 10,000 rpm and washed with chloroform for five times to completely remove the free PLA that was not grafted on the surface of HA-HMDI-EG. The HA-g-PLA was dried at 50 °C for 24 h in a vacuum oven. Three modified HA samples (HA-g-PLA(s), HA-g-PLA(m), and HA-g-PLA(l)) with different grafting degrees were prepared by the reaction between 1 g, 2 g, and 4 g of lactide and the 2 g of HA-HMDI-EG, respectively.

In addition, further experiment was performed to compare the amount of grafted PLA. Two grams of lactide were directly grafted on 2 g of HA (HA-g-PLA(d)) without coupling agents, and the other experimental procedure was same as above.

### 2.3. Preparation of Composites

The PLA/HA and PLA/HA-g-PLA composites were prepared as follows. Pre-weighed dried HA or HA-g-PLA particles were uniformly suspended in 70 folds (in weight) chloroform with sonication and stirring. Furthermore, the suspension was added into a 5 wt% PLA/chloroform solution to achieve the HA content of 5, 10, and 15 wt% in the composites. The mixtures were precipitated in an excess of ethanol, and the composites were dried in a vacuum-oven at 50 °C for 24 h to remove the residual solvent.

The PLA/PGA fiber composites were prepared as follows. Pre-weighed PGA fibers were suspended in chloroform with sonication. The ends of PGA fibers were held by the metal holders to keep the alignment parallel during sonication. Continuous PGA fibers were used to efficiently reinforce the composites. Additionally, a 5 wt% PLA/chloroform solution was added into the suspension to achieve the PGA fiber content of 10, 20, and 30 wt% in the composites. The mixture was dried at room temperature for 12 h first and at 50 °C for 24 h to remove the solvent.

In addition, HA or HA-g-PLA particles were uniformly suspended in chloroform, and added into PLA/chloroform solution in the same process as above. The PLA/HA mixtures in chloroform were added into the PLA/PGA fiber suspension in chloroform to achieve the HA content of 10 wt% and PGA fiber content of 30 wt% in the composites for PLA/PGA fiber/HA and PLA/PGA fiber/HA-g-PLA composites. The composites were dried at 50 °C for 24 h in a vacuum oven.

The composites were labelled by their compositions. The number after fillers denoted the amount of the components in wt%. For example, PLA/PGA fiber30/HA10 referred to a sample of PLA composite with 30 wt% of PGA fibers and 10 wt% of HA.

### 2.4. Characterization and Measurement

The chemical structures of each filler were analyzed by Fourier transform infrared (FT-IR, Bruker, TENSOR27, Billerica, MA, USA) spectroscopy with an attenuated total reflection accessory in the range from 4000 to 500 cm^−1^. The chemical bonds of each filler were analyzed by X-ray photoelectron spectroscopy (XPS, ThermoFisher Scientific, K-alpha+, Waltham, MA, USA) over the wide scanning energy range from 0 to 1350 eV with a pass energy of 200 eV and the high sensitivity spectrum of N1s with a pass energy of 40 eV. The amount of grafted materials on HA surface was determined by thermogravimetric analysis (TGA, TA Instruments, Discovery TGA, New Castle, DE, USA). The measurements were performed under nitrogen gas flow at a rate of 20 °C/min from room temperature to 100 °C, an isothermal process at 100 °C for 10 min, and a rate of 10 °C/min from 100 °C to 700 °C. The molecular weights of free PLA were analyzed by gel permeation chromatography (GPC, ThermoFisher Scientific, Ultimate 3000, Waltham, MA, USA). THF was used as solvent, and the molecular weights were calibrated with polystyrene standards. The morphological analysis of the composites was examined using field emission scanning electron microscope (FE SEM, Carl Zeiss, SUPRA 55VP, Oberkochen, Germany). The fracture surface was obtained by breaking the specimen after immersion in liquid nitrogen for 1 min.

The specimens for measuring tensile properties and flexural properties were prepared using hot-press (Carver, 3925, Wabash, IN, USA). Hot-press was performed at 180 °C. The tensile properties of the composites were determined using universal testing machine (UTM, Lloyd, LR10K, West Sussex, UK) at 25 °C and at the crosshead speed of 1 mm/min according to ISO 527-2 type 5A. The flexural properties of the composites were determined using the UTM at 25 °C and at the crosshead speed of 2 mm/min according to ISO 178.

For the bioactivity test, thin plate-shaped specimens prepared by the injection molding (Bautek, BA-915A, Gyeonggi-do, Korea) were immersed in PP bottles containing simulated body fluid (SBF) solution or phosphate buffer saline (PBS, pH 7.4) at 37 °C for various periods of time. A series of brief evacuation–repressurization cycles were carried out to force the solution into the pores of the composites until no air bubbles emerged from the composite surface. At the end of each immersing period, specimens were washed thoroughly with distilled water and dried at 50 °C for 48 h in a vacuum oven, and weighed.

## 3. Results and Discussion

### 3.1. Grafting of PLA on the Surface of HA

**Figure 1:**
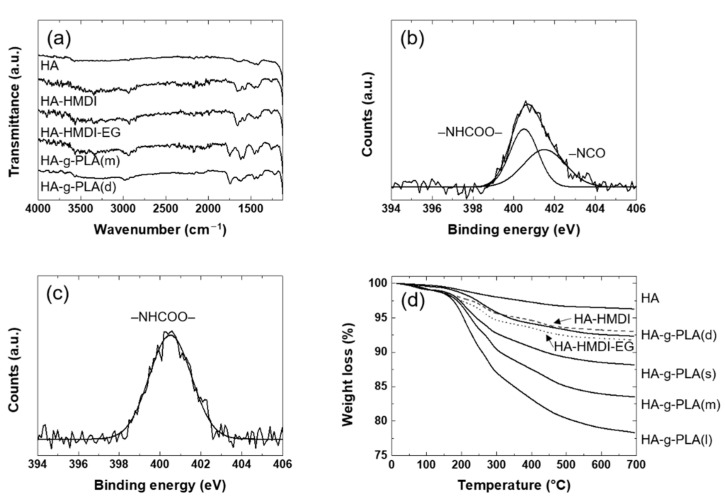
(**a**) FT-IR, (**b**,**c**) XPS for N1s, and (**d**) TGA of surface-grafted hydroxyapatite (HA): (**b**) HA-Hexamethylene diisocyanate (HMDI); (**c**) HA-HMDI-ethylene glycol (EG).

To identify the chemical reactions on the HA surface, the FT-IR spectra of the HA reacted in each step were investigated. In Figure 1a, the spectrum of HA-HMDI was similar to that of HA except for the characteristic peaks caused by HMDI. The spectrum exhibited the new peaks at 1664, 1570, and 2930 cm^−1^ due to the stretching of carbonyl groups, the bending of –NH in urethane bonds, and the –CH_2_ stretching in the HMDI, respectively. These were due to the reaction between the surface –OH groups of HA and isocyanate groups of HMDI. Similar results were observed in the HA reacted with the isocyanate groups [28]. The reaction between HA-HMDI and EG could not be confirmed by the FT-IR because the spectrum of HA-HMDI-EG was almost the same with that of HA-HMDI. The chemical reaction between HA-HMDI and EG were revealed by XPS as shown in Figure 1b,c. In N1s spectra of HA-HMDI, the remaining isocyanate groups appeared at 401.6 eV. The peak at 400.5 eV was assigned to the urethane bonds. The same bands were observed for the expanded graphite reacted with the isocyanate groups [29]. The atomic ratio of the urethane groups to the isocyanate groups was almost 1:1, indicating that side reactions including dimerization of HMDI and reaction between HA-HMDI and the impurity such as H_2_O were avoided [30,31]. In N1s spectra of HA-HMDI-EG, the peak of the isocyanate groups at 400.5 eV disappeared after additional urethane bonds were formed between the isocyanate groups of HA-HMDI and hydroxyl groups of EG. It was confirmed by FT-IR that PLA was grafted to HA-HMDI-EG. The spectrum of HA-g-PLA(m) exhibited a new characteristic peak at 1747 cm^−1^ due to the stretching of carbonyl groups in ester bonds, which was formed by ring-opening polymerization (ROP) of lactide on the hydroxyl groups of HA-HMDI-EG. The same peak was observed in a study of grafting PLA to HA [32]. In the spectrum of HA-g-PLA(d), the stretching of carbonyl groups in ester bonds also appeared at 1743 cm^−1^ due to the PLA grafted to the surface –OH groups of HA.

By sequentially reacting HMDI and EG to HA, the end groups of the HA were changed to hydroxyl groups capable of efficient ROP of lactide. In order to estimate the amount of coupling agents and PLA grafted to the HA, the TGA of the HA reacted in each step were investigated as shown in Figure 1d. The amounts of HMDI bound to HA and EG reacted with HA-HMDI were 3.3% (0.21 mmol/g(HA)) and 1.2% (0.18 mmol/g(HA)), respectively. The difference in the amount of reacted materials was interpreted as an error that might occur during TGA measurement. It could also be explained by the fact that the atomic ratio of the urethane groups to the isocyanate groups was almost 1:1 in the N1s spectra of XPS of HA-HMDI. The amount of grafted PLA by the direct grafting and the use of coupling agents was 4% and 3.6–13.4%, respectively. When comparing HA-g-PLA(d) and HA-g-PLA(m), the same experimental amount of lactide, it was clear that the hydroxyl end groups of HA-HMDI-EG caused the ROP of lactide more easily. PLA was grafted about twice as much in HA-g-PLA(m) than in HA-g-PLA(d).

The change of the length and frequency of PLA surrounding HA could affect the interface between the matrix PLA and the HA. Since the mechanical properties of polymer composites were critically affected by the interface, the quantitative analysis of the grafted PLA should be investigated in detail. In order to determine the length of the PLA grafted to HA, the molecular weight of the free PLA was measured by GPC in the same manner as in other study [19]. Free PLA was formed by impurities such as residual water, and was not bound to HA. In a study, the molecular weight of the free polymer was similar to that of the polymer obtained by cleaving the bonds between the particles and the grafted polymers [33]. In this study, it was also assumed that the molecular weight of PLA grafted to HA was similar to that of free PLA. The molecular weight of PLA grafted through the coupling agents with different grafted chain length (HA-g-PLA(s), HA-g-PLA(m), and HA-g-PLA(l)) and PLA grafted directly (HA-g-PLA(d)) is shown in Table 1. In HA-g-PLA(d) and HA-g-PLA(m), the same experimental amount of lactide, the molecular weight of free PLA was 8 times smaller in HA-g-PLA(m). This was because the introduction of coupling agents increased the amount of reactive initiator by changing the end groups of HA to hydroxyl groups, thereby decreasing the monomer to initiator ratio. With the characteristic ratio of PLA of 11.8, the end-to-end distance of the grafted polymer was calculated at theta condition [34]. The area occupied by each grafted PLA chain was calculated based on the TGA results, molecular weight of the grafted PLA, Avogadro’s number, and the specific surface area of HA of 80 m^2^/g. From these results, it was shown that relatively large PLA was grafted at low density in HA-g-PLA(d). When PLA was grafted through coupling agents, smaller polymers were distributed at higher density. Despite the change in the experimental amount of lactide, the density of the grafted PLA was almost the same. The molecular weight of the grafted PLA was only changed. This could be explained by the fact that the amount of monomer was only changed while the amount of initiator remained the same.

### 3.2. Mechanical Properties and Morphology

Table 2 showed the tensile properties and the flexural properties of PLA, PLA/HA, PLA/HA-g-PLA, and PLA/PGA fiber composites. Young’s modulus of the PLA/HA increased with increasing HA content compared to that of PLA. Elongation at break of the PLA/HA decreased compared to that of PLA due to the poor interfacial adhesion between HA and matrix PLA. This resulted in decrease in tensile strength of the PLA/HA compared to that of PLA. The interfacial adhesion between the HA and matrix PLA was improved by grafting PLA to HA. Elongation at break of PLA/HA-g-PLA(m) increased compared to that of PLA/HA for all composition due to the entanglements between the grafted PLA chains and the matrix PLA chains. The tensile strength of PLA/HA-g-PLA(m) increased compared to that of PLA up to 10 wt% of HA-g-PLA(m). The Young’s modulus and tensile strength of PLA/HA-g-PLA(m)10 increased by 26% and 5% than those of PLA resin, respectively.

The tensile strength of PLA/HA-g-PLA composites with different PLA grafted to HA was investigated because the tensile strength of the composites would be affected by the interface between the filler and the matrix. The tensile strength of the PLA composites containing 10 wt% of HA-g-PLA using the coupling agents all increased compared to that of PLA resin due to the improved interfacial adhesion. Regardless of the length of the grafted PLA, the interface was improved to a similar level due to the entanglements between all the same PLA chains. Since the interfacial strength was the same, the difference in tensile strength of the composites containing HA-g-PLA using the coupling agents was within the error range.

On the other hand, the interface area between the matrix PLA and the grafted PLA in PLA/HA-g-PLA(d) was not as large as that in PLA/HA-g-PLA(s),(m),(l) because the surface of HA-g-PLA(d) was not sufficiently covered with PLA. The tensile strength of PLA/HA-g-PLA(d) decreased compared to that of PLA resin because the interfacial adhesion was not sufficiently improved. The same result was observed for the PLA composites containing the directly grafted HA [20]. The interface between the HA and the matrix PLA was changed as the environment such as the length and frequency of the grafted PLA varied. Although it might have an important effect on the mechanical properties of the composites, it has not been investigated in detail in the other PLA/HA-g-PLA papers [19,20,35,36]. Since the difference in tensile strength of PLA composites containing HA-g-PLA(s), HA-g-PLA(m), and HA-g-PLA(l) was very small, the subsequent experiments using the HA grafted by PLA were fixed with HA-g-PLA(m).

Although the tensile strength of PLA/HA-g-PLA(m)10 increased compared to that of PLA resin, the improvement was not sufficient. The tensile strength of the composites was significantly improved by manufacturing PLA composites containing unidirectionally aligned PGA fibers. Efficient stress transfer occurred between the aligned PGA fiber and the PLA matrix through bridging effect under the tensile deformation [37]. The tensile strength of PLA/PGA fiber composites increased with increasing PGA fiber content, and the tensile strength of PLA/PGA fiber30 increased by 109% compared to that of PLA.

**Figure 2:**
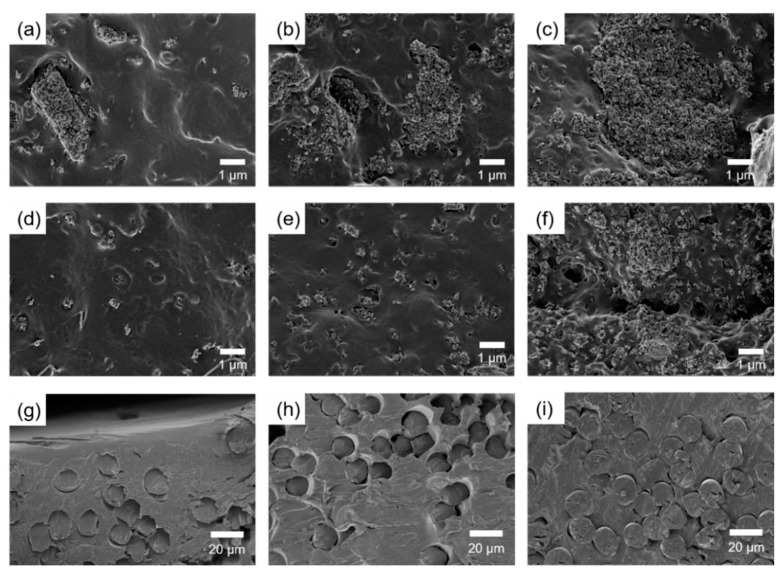
SEM images of fracture surface of (**a**) PLA/HA5, (**b**) PLA/HA10, (**c**) PLA/HA15, (**d**) PLA/HA-g-PLA(m)5, (**e**) PLA/HA-g-PLA(m)10, (**f**) PLA/HA-g-PLA(m)15, (**g**) PLA/PGA fiber10, (**h**) PLA/PGA fiber20, and (**i**) PLA/PGA fiber30.

The mechanical properties of the composites were affected not only by interfacial adhesion between the matrix and the filler but also by dispersion of the filler. The morphology of the fracture surface of the composites was shown in Figure 2. The non-grafted HA particles agglomerated at several micrometers even at 5 wt% in the composites. The chloroform used to make the PLA solution was not suitable for HA dispersion due to its low polarity. This indicated that the HA particles separated through ultrasonic treatment were agglomerated again during the fabrication of composites due to their high surface energy. Due to the aggregation of HA, specimen failure occurred early, resulting in a decrease in the tensile strength of the PLA/HA composites.

Compared to the non-grafted HA, submicron-sized HA-g-PLA(m) was observed in the composites with up to 10 wt% of HA-g-PLA(m). It could be explained that the cohesion between the HA particles was reduced by grafting of PLA onto the HA surface. Young’s modulus of PLA/HA-g-PLA(m) was higher than that of PLA/HA because HA-g-PLA(m) was dispersed with less agglomeration. Since the Young’s modulus was determined at the very initial deformation, Young’s modulus of the composites was greatly influenced by the dispersion of the filler. In a previous study, the grafted HA acted as heterogeneous nucleation sites in the matrix PLA, increasing the crystallinity of the matrix [35]. The mechanical properties of the composites would be improved by the increase in the crystallinity of the PLA matrix through evenly dispersed HA-g-PLA(m). At 15 wt%, HA-g-PLA(m) also agglomerated, resulting in decrease in the tensile strength of PLA/HA-g-PLA(m)15 compared to that of PLA due to the early fracture. As shown in Figure 2g–i, the PGA fibers did not aggregate in the PLA matrix, especially even at 30 wt%, because of the similar surface energy between PLA and PGA [38]. Through the evenly distributed PGA fibers, stress transfer between the PGA fibers and the PLA matrix could occur efficiently.

As the stress state experienced by the spine bone was close to bending rather than tensile deformation, flexural properties of the composites were more important for spinal implant materials. The tendency of changes in flexural properties such as flexural modulus, flexural strength, and flexural strain at break was the same to that in tensile properties. The flexural strength of PLA/HA-g-PLA(m)10 and PLA/PGA fiber30 increased by 6% and 39%, respectively, compared to that of PLA resin. The degrees of increase were not as large as in the tensile strength. This could be explained by the fact that bending deformation was a mixture of tension and compression and that unidirectional long fibers were not as effective in compression as in tension for reinforcing polymeric materials.

**Figure 3:**
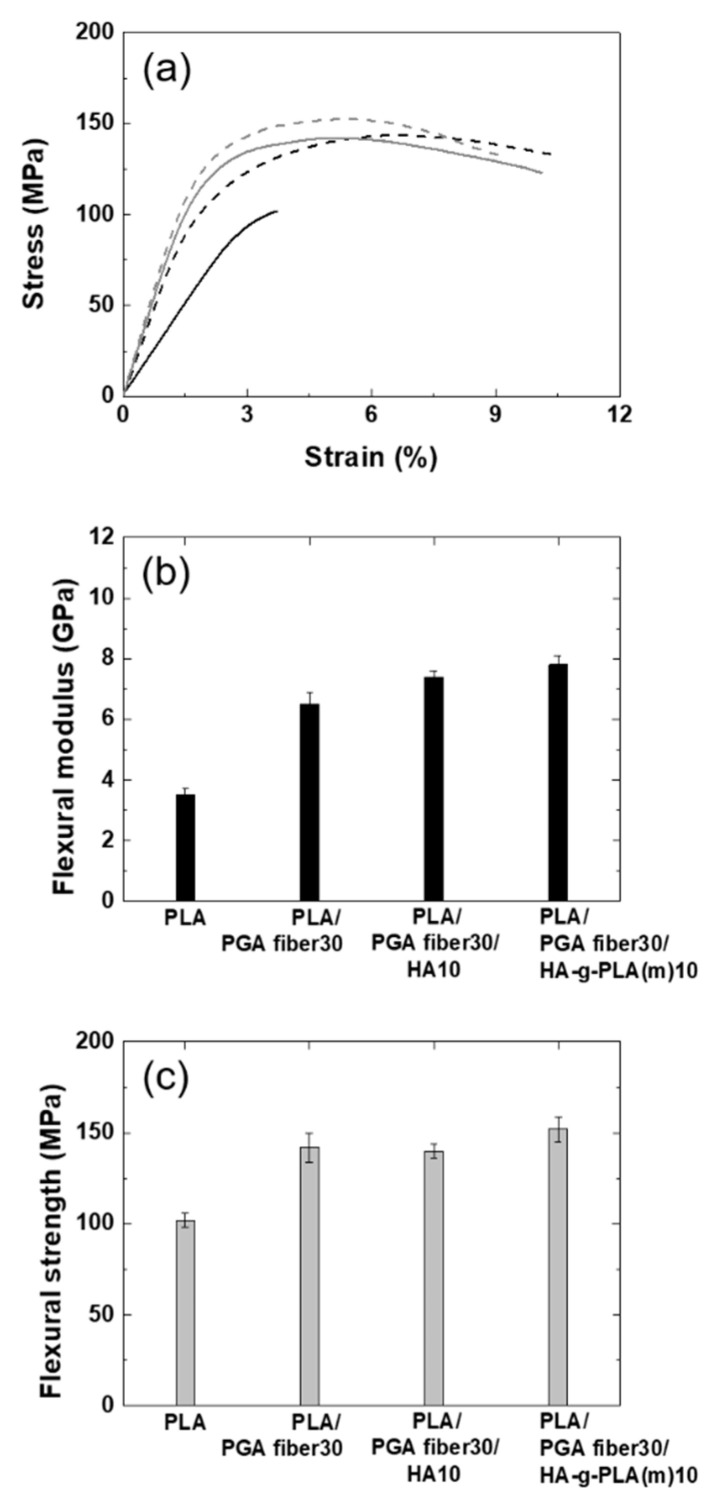
(**a**) Flexural stress–strain curves, (**b**) flexural modulus, and (**c**) flexural strength of PLA and its composites: PLA (black solid line); PLA/PGA fiber30 (black dash line); PLA/PGA fiber30/HA10 (gray solid line); PLA/PGA fiber30/HA-g-PLA(m)10 (gray dash line).

For a material to be used in spinal implants, the flexural strength has been desired to be similar to that of a human spine bone [39]. The flexural strength of the composites could be greatly improved by introducing the unidirectionally aligned PGA fibers. However, the processability of composites exceeding 30 wt% of PGA fibers might be poor, and increasing the PGA fiber content could lead to rapid biodegradation of the composites. In order to increase the flexural strength of the PLA composites containing HA, HA should be used in a range where HA did not aggregate. Therefore, 10 wt% of HA and 30 wt% of aligned PGA fiber were added to the composites. To investigate the role of each filler in the composites containing two fillers, the flexural properties of PLA, PLA/PGA fiber30, PLA/PGA fiber 30/HA10, and PLA/PGA fiber30/HA-g-PLA(m)10 were investigated as shown in Figure 3. Flexural modulus of PLA/PGA fiber30/HA10 increased by 14% compared to that of PLA/PGA fiber30. However, the flexural strength of PLA/PGA fiber30/HA10 was almost the same as that of PLA/PGA fiber30 due to poor interfacial adhesion between HA and PLA matrix. Flexural strength increased further to 152 MPa only in PLA/PGA fiber30/HA-g-PLA(m)10.

Hong et al. showed that the flexural strength of PLA composites containing 4 wt% PLA-grafted HA was about 4% higher than that of PLA resin, whereas the flexural strength of PLA/PGA fiber30/HA-g-PLA (m)10 increased by 49% compared to that of PLA resin [20]. Also, this value was similar to the average value of the flexural strength of distal part of the human compact bone known as 104–192 MPa [11,40]. The improvements in the flexural strength of biodegradable polymers and HA composites to levels similar to that of human bones have not been reported to the best of our knowledge. Compared to titanium and magnesium, the mechanical properties of PLA/PGA fiber30/HA-g-PLA(m)10 composite were more suitable for spinal implant materials. The modulus of titanium and magnesium was too high compared to that of the human compact bone, which was 9.8–13.1 GPa [40]. On the other hand, the flexural modulus of the PLA/PGA fiber30/HA-g-PLA(m)10 composite was 7.8 GPa, which could avoid the stress shielding effect.

### 3.3. Bioactivity

**Figure 4:**
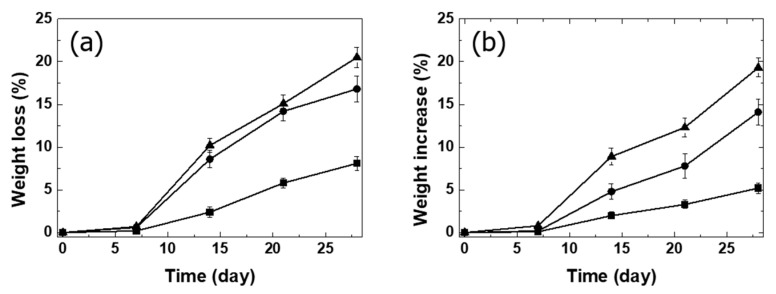
(**a**) Weight loss by phosphate buffer saline (PBS) and (**b**) weight increase by simulated body fluid (SBF) solution of the composites: ■ PLA/PGA fiber30; ● PLA/PGA fiber30/HA10; ▲ PLA/PGA fiber30/HA-g-PLA(m)10.

In order to be applied to spinal implant materials, it was necessary to improve the bioactivity as well as the flexural strength of PLA composites. To compare the bioactivity of the composites, the behaviors of PLA/PGA fiber30, PLA/PGA fiber30/HA10, and PLA/PGA fiber30/HA-g-PLA(m)10 composites in SBF solution were monitored. The composites were immersed in SBF solution, and the rate and the amount of growth of new apatite on the composite surface were recorded [41,42]. The calculation of weight increase by apatite growth was based on the weight loss of the specimen in PBS over the same period [43]. Weight loss due to the hydrolysis of biodegradable polymers and the fall-off of HA particles was determined by the surface wettability of H_2_O [16]. The surface of PLA/PGA fiber30/HA-g-PLA(m)10 was more hydrophilic than that of other composites due to the HA particles located on the composite surface. Weight loss and weight increase were greater in PLA/PGA fiber30/HA-g-PLA(m)10 than in other composites as shown in Figure 4. The surface area covered with HA particles was smaller in PLA/PGA fiber30/HA10 than PLA/PGA fiber30/HA-g-PLA(m)10 due to the HA aggregation. In PLA/PGA fiber30/HA10, the HA particles easily fell off due to their poor interfacial adhesion. This resulted in a small difference in weight loss between PLA/PGA fiber30/HA10 and PLA/PGA fiber30/HA-g-PLA(m)10, but greater difference in weight increase. The PLA/PGA fiber30 was the most hydrophobic and the PBS and SBF solution were difficult to contact with the specimen surface.

**Figure 5:**
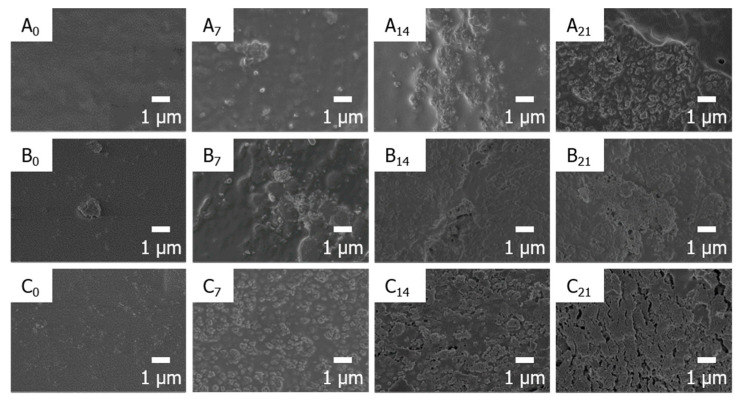
Surface SEM images of the composites before and after apatite growth by immersing PLA/PGA fiber30 (**A**), PLA/PGA fiber30/HA10 (**B**), and PLA/PGA fiber30/HA-g-PLA(m)10 (**C**) in SBF solution. The subscript number represents the immersing days of the composites.

The SEM micrographs of the composites showed that the apatite began to precipitate on the surface of PLA and its composites as shown in Figure 5. As the soaking time increased, new apatite was observed on the surface of the composites. The new apatite grew most uniformly in the PLA/PGA fiber30/HA-g-PLA(m)10 because the new apatite is nucleated based on the HA present on the composite surface [44]. In PLA/PGA fiber30/HA10, new apatite grew irregularly due to the HA aggregation and the fall-off of HA particles during hydrolysis. Apatite precipitation was observed most slowly in PLA/PGA fiber30 specimens due to lack of nucleation sites and hydrophobicity.

## 4. Conclusions

By introducing coupling agents into the grafting reaction, a larger amount of PLA was grafted to the HA. When PLA was grafted through a coupling agent, the HA surface could be sufficiently covered with PLA, resulting in the improvement of interface between matrix PLA and HA. The dispersion of the HA was also improved by grafting PLA with coupling agents. The tensile strength and flexural strength of PLA/HA-g-PLA(m) were higher than those of PLA/HA or PLA resin. PLA composites with PGA fibers aligned in one direction significantly improved mechanical properties of the composites. In PLA/PGA fiber/HA-g-PLA(m), the mechanical properties and the rate of new apatite growth on the surface of the composites by immersing the specimens in SBF were significantly improved compared to those of PLA resin. All these results indicated that the PLA/PGA/HA-g-PLA could be promising for spinal implant materials.

## Figures and Tables

**Table 1:** Quantitative analysis of HA-g-PLA.

	Graft Amount (%)	Free Polymer Molecular Weight (g/mol)	End-to-End Distance ^1^ (nm)	Area per Grafted Chain (nm^2^)
HA-g-PLA(d)	4.0	61,500	25	204
HA-g-PLA(s)	3.6	3600	6	13
HA-g-PLA(m)	8.2	7800	9	13
HA-g-PLA(l)	13.4	13,900	12	13

^1^ At theta condition.

**Table 2:** Mechanical properties of PLA and its composites.

	Young’s Modulus (GPa)	Tensile Strength (MPa)	Elongation at Break (%)	Flexural Modulus (GPa)	Flexural Strength (MPa)	Flexural Stain at Break (%)
PLA	3.1	74	3.3	3.5	102	3.9
PLA/HA5	3.3	63	2.1	3.9	84	2.3
PLA/HA10	3.7	56	1.7	4.4	77	1.9
PLA/HA15	4.1	43	1.2	4.9	69	1.5
PLA/HA-g-PLA(m)5	3.4	76	2.4	4.0	105	3.4
PLA/HA-g-PLA(m)10	3.9	78	2.2	4.6	108	3.2
PLA/HA-g-PLA(m)15	4.3	66	1.6	5.0	93	2.1
PLA/HA-g-PLA(d)10	3.7	68	2.0	NA	-	-
PLA/HA-g-PLA(s)10	3.9	77	2.2	NA	-	-
PLA/HA-g-PLA(l)10	3.8	76	2.3	NA	-	-
PLA/PGA fiber10	3.5	99	24.8	4.6	118	- ^1^
PLA/PGA fiber20	3.9	130	22.6	5.6	130	-
PLA/PGA fiber30	4.2	155	25.4	6.5	142	-

^1^ Not broken during flexural test.

## Data Availability

The data presented in this study are available on request from the corresponding author.
